# The genome sequence of the Alder leaf beetle, *Agelastica alni *(Linnaeus, 1758)

**DOI:** 10.12688/wellcomeopenres.24286.1

**Published:** 2025-05-20

**Authors:** Danaë Vassiliades, Michael F. Geiser, Maxwell V. L. Barclay, Will Bayfield Farrell, Joana Cristóvão, Keita Matsumoto, Liam M. Crowley, Natural History Museum Genome Acquisition Lab, University of Oxford and Wytham Woods Genome Acquisition Lab, Darwin Tree of Life Barcoding collective, Wellcome Sanger Institute Tree of Life Management, Samples and Laboratory team, Wellcome Sanger Institute Scientific Operations: Sequencing Operations, Wellcome Sanger Institute Tree of Life Core Informatics team, Tree of Life Core Informatics collective, Darwin Tree of Life Consortium

**Affiliations:** 1 Natural History Museum, London, England, UK; 2 Department of Biology, University of Oxford, Oxford, England, UK

**Keywords:** Agelastica alni, Alder leaf beetle, genome sequence, chromosomal, Coleoptera

## Abstract

We present a genome assembly from a specimen of *Agelastica alni* (Alder leaf beetle; Arthropoda; Insecta; Coleoptera; Chrysomelidae). The genome sequence has a total length of 692.27 megabases. Most of the assembly (98.71%) is scaffolded into 12 chromosomal pseudomolecules, including the X sex chromosome. The mitochondrial genome has also been assembled, with a length of 17.39 kilobases. Gene annotation of this assembly on Ensembl identified 13,498 protein-coding genes.

## Species taxonomy

Eukaryota; Opisthokonta; Metazoa; Eumetazoa; Bilateria; Protostomia; Ecdysozoa; Panarthropoda; Arthropoda; Mandibulata; Pancrustacea; Hexapoda; Insecta; Dicondylia; Pterygota; Neoptera; Endopterygota; Coleoptera; Polyphaga; Cucujiformia; Chrysomeloidea; Chrysomelidae; Galerucinae; Sermylini; Agelasticites; *Agelastica*; *Agelastica alni* (Linnaeus, 1758) (NCBI:txid131577)

## Background

*Agelastica alni* (Linnaeus, 1758) (Coleoptera: Chrysomelidae: Galerucinae) is a medium-sized metallic blue leaf beetle found across the Palaearctic Region. In the UK, it can be easily distinguished from somewhat morphologically similar species in the genus *Altica* (Galerucinae: Alticini), but *Alitca members* have enlarged hind femora and lack a pronotal groove. Apart from the superficial resemblance to *Altica, Agelastica alni* is unmistakeable among the fauna of Britain and Ireland.

*Agelastica* Chevrolat, 1836 is a small genus within the Galerucinae, comprising two valid species: *A. alni* and *A. coerulea* Baly, 1874 plus one subspecies, *A. alni glabra* (Fischer von Waldheim, 1842) ( Bezděk & Sekerka, 2024). *A. coerulea, A. alni* and *A. alni glabra* are found within the Palaearctic Region and form a complex of morphologically similar taxa distinguished mainly by proportions of the 3rd and 4th antennomeres and the shape of the anterior angles of the pronotum ( Bezděk, 2015; Warchałowski, 2010). *A. alni glabra* was cited as *A. orientalis* in the latest World Catalogue of Galerucinae ( Wilcox, 1971) and later as *A. alni orientalis* ( Beenen, 2010). Bezděk later synonymised *A. orientalis* with *A. alni glabra* ( Bezděk, 2015). Additionally, *A. cyanicollis* (Jacoby, 1884) and *A. lineata* Blackburn 1888 from Sumatra and Australia respectively, are still formally placed within *Agelastica* ( Wilcox, 1971) but do not seem to be relatives of the Palaearctic species and should be considered ’incertae sedis’. *Diacantha bimaculata* Bertoloni, 1868, listed as an *Agelastica* by Wilcox ( Wilcox, 1971), was later moved into *Hallirhotius* ( Beenen, 2020).

The current range of *A. alni* encompasses large parts of the Western Palaearctic, including Europe, Turkey and Iran ( Bezděk & Sekerka, 2024). *A. alni* is notable in the British fauna for its recent re-emergence after being declared locally extinct in 1987 ( Shirt, 1987) owing to a dearth of records post-1900. Though some sparse records between 1940 and 1958 were later noted ( Allen, 1994; Cox, 2007; Hyman & Parsons, 1992; Lewis, 2004), these are likely to be fleeting reintroductions which did not lead to reestablishment of the species ( Stenhouse, 2019). However, after an explosion of records in the early 21st century originating in northwest England, the beetle has firmly re-established itself in Britain, with populations spreading southwards ( iRecord, 2024; Stenhouse, 2019). DNA barcoding of individuals from across the current British range has confirmed that the *Agelastica* present in Britain today are indeed *A. alni* and not *A. coerulea* ( Vassiliades, 2024).

As the common name ‘Alder Leaf Beetle’ suggests, both adults and larvae feed primarily on alders, particularly *Alnus glutinosa* and *A. incana* ( Stenhouse, 2019) *. A. alni* has also been seen to utilise other species in the Betulaceae ( Cox, 2007; Ramsay, 2009) including hazel ( *Corylus* spp.); on which it sometimes reaches commercial pest status in Turkey ( Sezen & Demirbağ, 2006). The species has additionally been observed feeding on goat willow *Salix caprea* ( Cox, 2007), and adults of the current British populations may be more polyphagous than previously described in past literature. Both adults and larvae of *A. alni* often occur in large numbers on *Alnus*, and as a result can cause significant and unsightly defoliation of host trees. As such, the beetle is considered a garden pest in the UK ( RHS, 2024).

**Figure 1.**
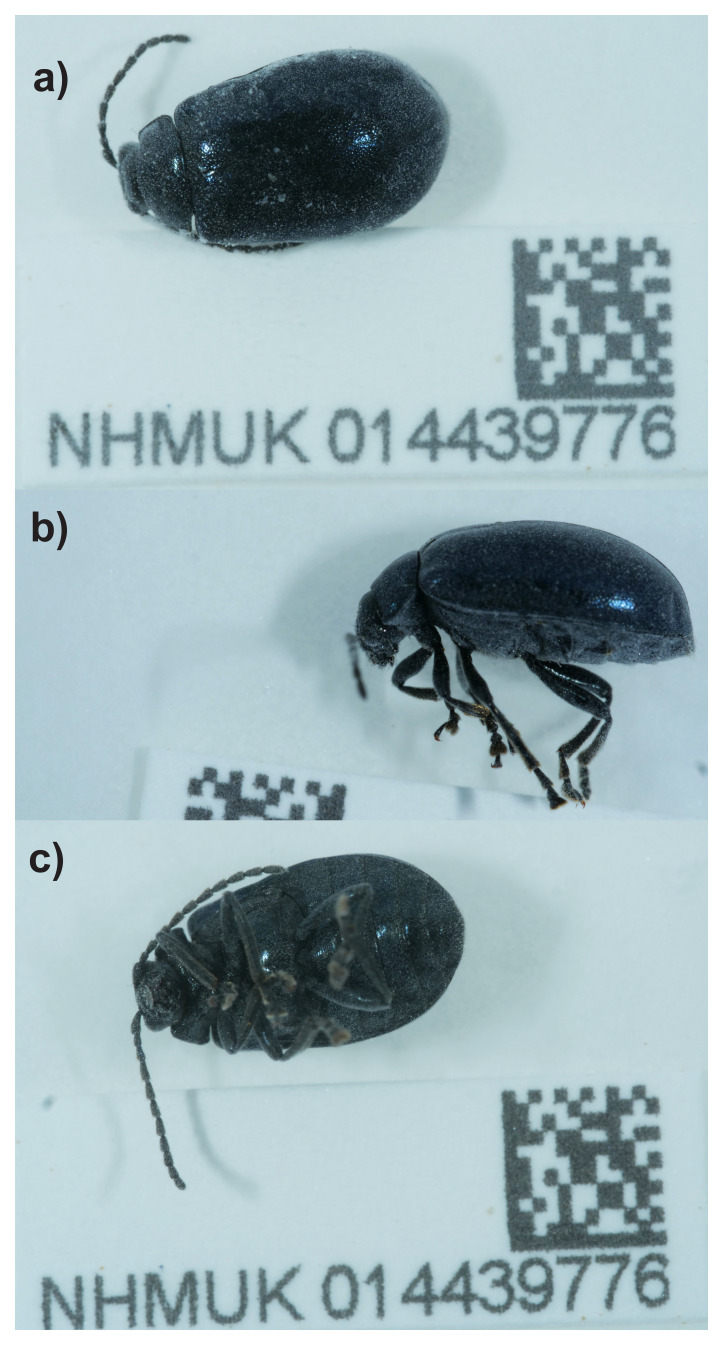
Photograph of the *Agelastica alni* (icAgeAlni6) specimen used for genome sequencing.

Here we present a chromosomally complete genome sequence for *Agelastica alni*, based on a specimen from Bookham Common, Surrey, England, United Kingdom ( Figure 1).

## Genome sequence report

### Sequencing data

The genome of a specimen of *Agelastica alni* ( Figure 1) was sequenced using Pacific Biosciences single-molecule HiFi long reads, generating 25.32 Gb from 2.21 million reads, which were used to assemble the genome. GenomeScope analysis estimated the haploid genome size at 799.44 Mb, with a heterozygosity of 0.08% and repeat content of 43.96%. These estimates guided expectations for the assembly. Based on the estimated genome size, the sequencing data provided approximately 30 coverage. Hi-C sequencing produced 141.16 Gb from 934.86 million reads, and was used to scaffold the assembly. RNA sequencing data were also generated and are available in public sequence repositories. Table 1 summarises the specimen and sequencing details.

**Table 1.** Specimen and sequencing data for *Agelastica alni*.

Project information			
**Study title**	Agelastica alni (alder leaf beetle)		
**Umbrella BioProject**	PRJEB60008		
**Species**	*Agelastica alni*		
**BioSpecimen**	SAMEA14448393		
**NCBI taxonomy ID**	131577		
Specimen information			
**Technology**	**ToLID**	**BioSample accession**	**Organism part**
**PacBio long read sequencing**	icAgeAlni6	SAMEA14448739	thorax
**Hi-C sequencing**	icAgeAlni4	SAMEA10979659	whole organism
**RNA sequencing**	icAgeAlni5	SAMEA10979660	whole organism
Sequencing information			
**Platform**	**Run accession**	**Read count**	**Base count (Gb)**
**Hi-C Illumina NovaSeq 6000**	ERR10908644	9.35e+08	141.16
**PacBio Sequel IIe**	ERR10906113	2.21e+06	25.32
**RNA Illumina NovaSeq 6000**	ERR10908645	7.39e+07	11.16

### Assembly statistics

The primary haplotype was assembled, and contigs corresponding to an alternate haplotype were also deposited in INSDC databases. The assembly was improved by manual curation, which corrected 59 misjoins or missing joins and removed three haplotypic duplications. These interventions decreased the scaffold count by 24.29% and increased the scaffold N50 by 18.46%. The final assembly has a total length of 692.27 Mb in 133 scaffolds, with 245 gaps, and a scaffold N50 of 66.31 Mb ( Table 2).

**Table 2.** Genome assembly data for *Agelastica alni*.

Genome assembly		
Assembly name	icAgeAlni6.2	
Assembly accession	GCA_950111635.2	
*Alternate haplotype accession*	*GCA_950106765.2*	
Assembly level for primary assembly	chromosome	
Span (Mb)	692.27	
Number of contigs	378	
Number of scaffolds	133	
Longest scaffold (Mb)	100.21	
Assembly metric	Measure	*Benchmark*
Contig N50 length	4.55 Mb	*≥ 1 Mb*
Scaffold N50 length	66.31 Mb	*= chromosome N50*
Consensus quality (QV)	Primary: 55.9; alternate: 56.3; combined: 56.1	*≥ 40*
*k*-mer completeness	Primary: 96.00%; alternate: 81.40%; combined: 98.11%	*≥ 95%*
BUSCO *	C:99.2%[S:97.7%,D:1.6%], F:0.2%,M:0.5%,n:2,124	*S > 90%; D < 5%*
Percentage of assembly mapped to chromosomes	98.71%	*≥ 90%*
Sex chromosomes	X	*localised homologous pairs*
Organelles	Mitochondrial genome: 17.39 kb	*complete single alleles*

* BUSCO scores based on the endopterygota_odb10 BUSCO set using version 5.5.0. C = complete [S = single copy, D = duplicated], F = fragmented, M = missing, n = number of orthologues in comparison.

**Figure 2.**
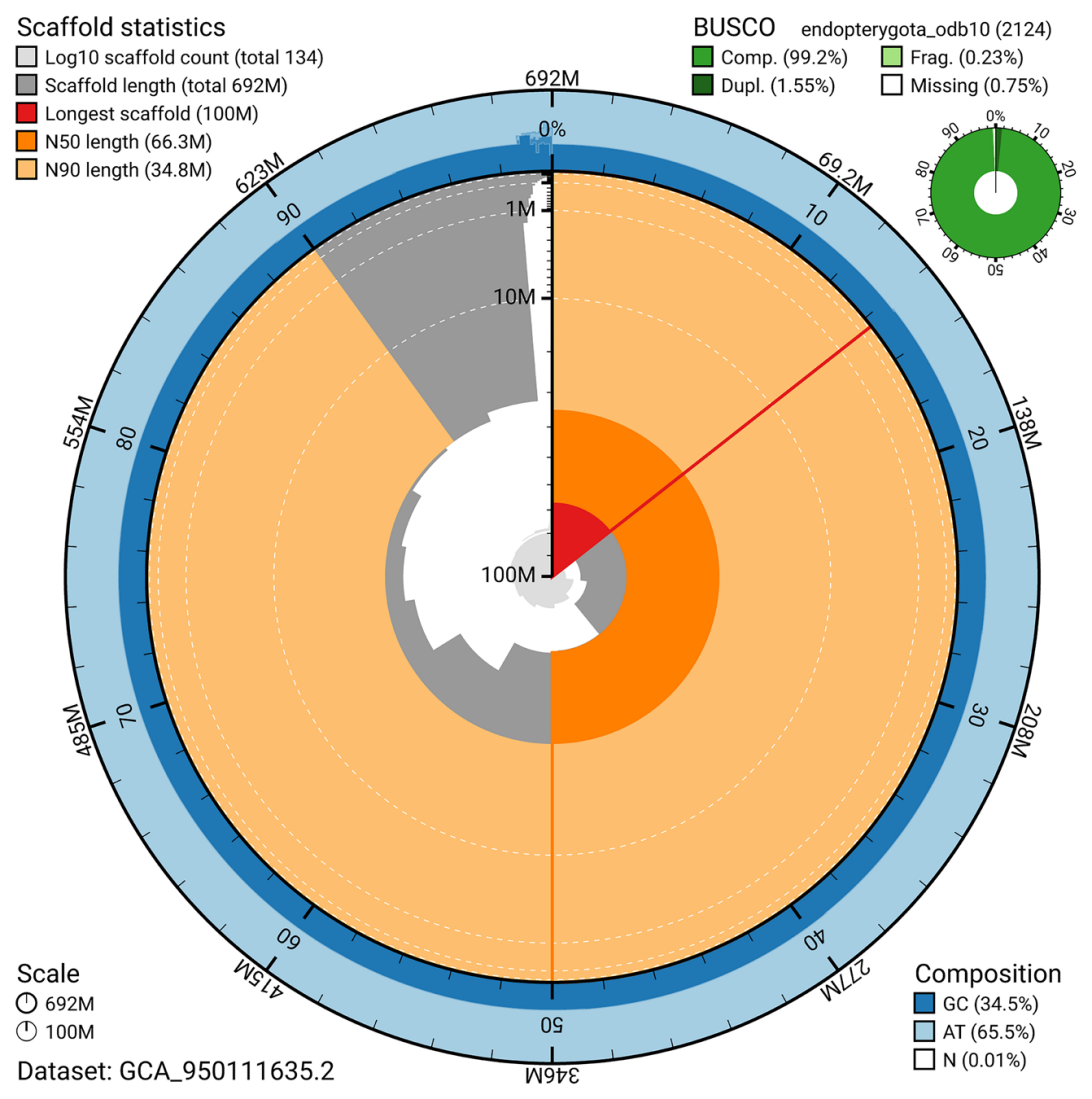
Genome assembly of *Agelastica alni*, icAgeAlni6.2: metrics. The BlobToolKit snail plot provides an overview of assembly metrics and BUSCO gene completeness. The circumference represents the length of the whole genome sequence, and the main plot is divided into 1,000 bins around the circumference. The outermost blue tracks display the distribution of GC, AT, and N percentages across the bins. Scaffolds are arranged clockwise from longest to shortest and are depicted in dark grey. The longest scaffold is indicated by the red arc, and the deeper orange and pale orange arcs represent the N50 and N90 lengths. A light grey spiral at the centre shows the cumulative scaffold count on a logarithmic scale. A summary of complete, fragmented, duplicated, and missing BUSCO genes in the endopterygota_odb10 set is presented at the top right. An interactive version of this figure is available at https://blobtoolkit.genomehubs.org/view/GCA_950111635.2/dataset/GCA_950111635.2/snail.

**Figure 3.**
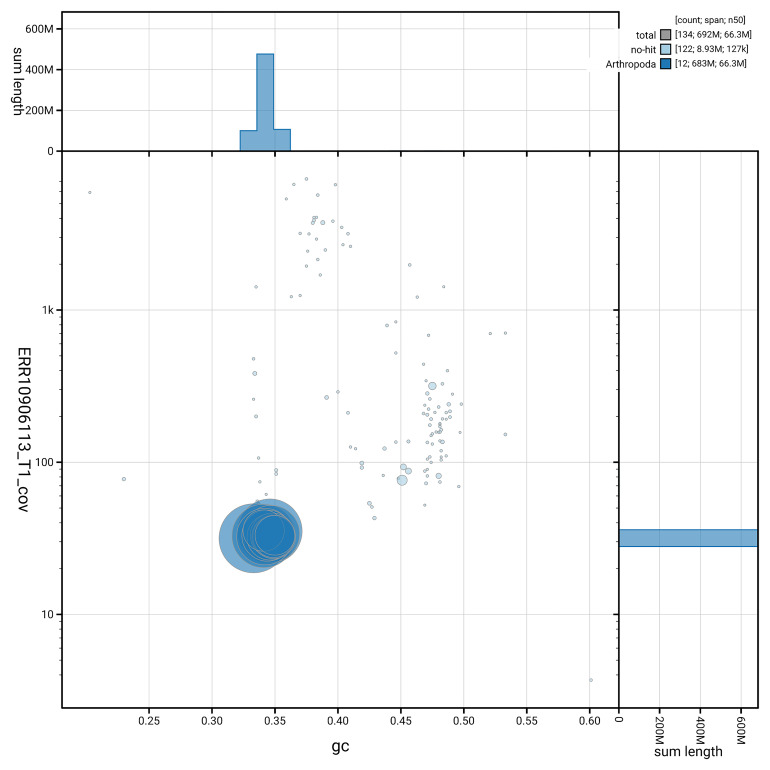
Genome assembly of *Agelastica alni*, icAgeAlni6.2: BlobToolKit GC-coverage plot. Blob plot showing sequence coverage (vertical axis) and GC content (horizontal axis). The circles represent scaffolds, with the size proportional to scaffold length and the colour representing phylum membership. The histograms along the axes display the total length of sequences distributed across different levels of coverage and GC content. An interactive version of this figure is available at https://blobtoolkit.genomehubs.org/view/GCA_950111635.2/dataset/GCA_950111635.2/blob.

**Figure 4.**
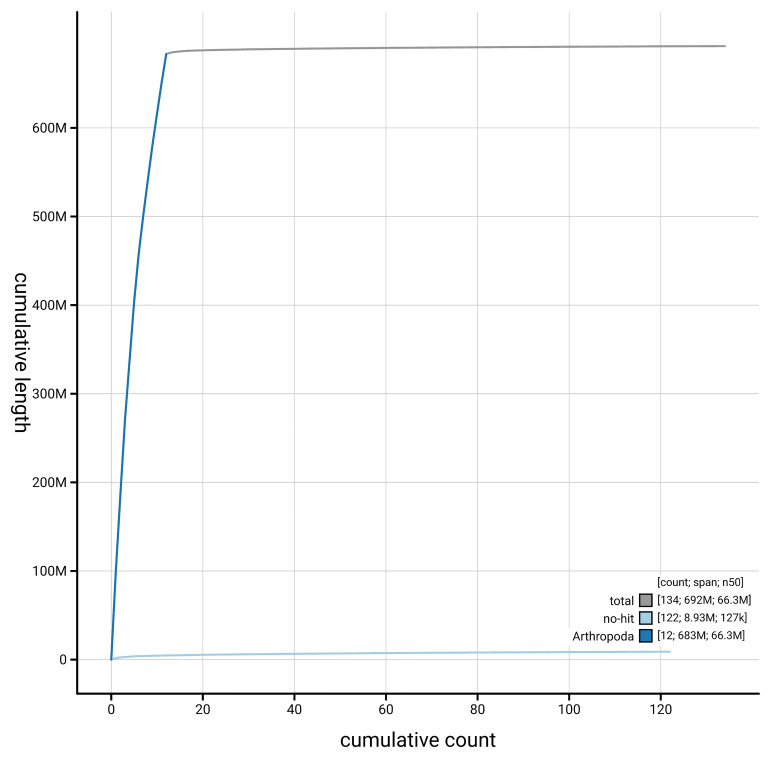
Genome assembly of *Agelastica alni,* icAgeAlni6.2: BlobToolKit cumulative sequence plot. The grey line shows cumulative length for all scaffolds. Coloured lines show cumulative lengths of scaffolds assigned to each phylum using the buscogenes taxrule. An interactive version of this figure is available at https://blobtoolkit.genomehubs.org/view/GCA_950111635.2/dataset/GCA_950111635.2/cumulative.

The snail plot in Figure 2 provides a summary of the assembly statistics, indicating the distribution of scaffold lengths and other assembly metrics. Figure 3 shows the distribution of scaffolds by GC proportion and coverage. Figure 4 presents a cumulative assembly plot, with separate curves representing different scaffold subsets assigned to various phyla, illustrating the completeness of the assembly.

**Figure 5.**
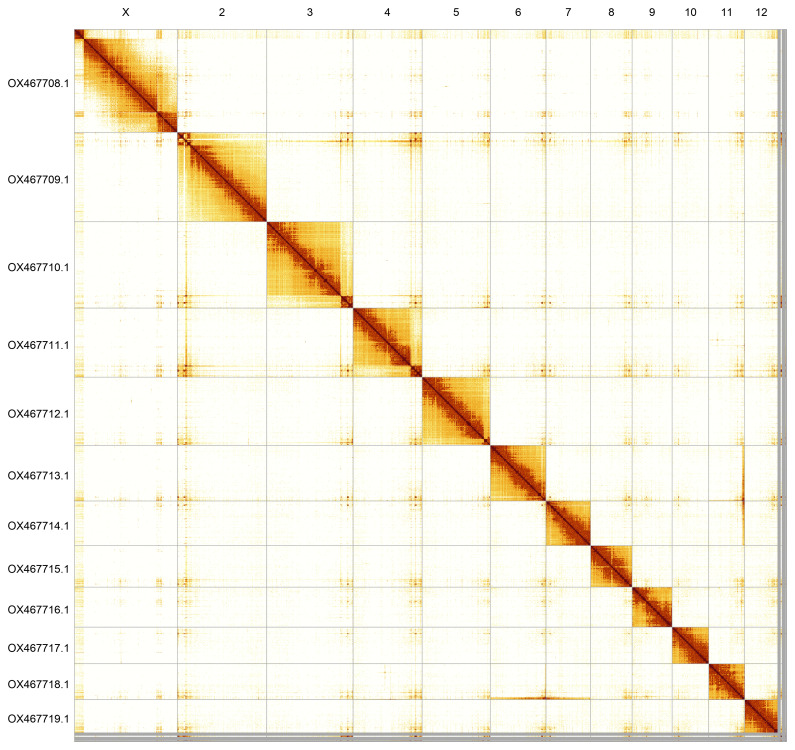
Genome assembly of *Agelastica alni*: Hi-C contact map of the icAgeAlni6.2 assembly, produced in PretextView. Chromosomes are shown in order of size from left to right and top to bottom.

Most of the assembly sequence (98.71%) was assigned to 12 chromosomal-level scaffolds, representing 12 autosomes and the X sex chromosome. These chromosome-level scaffolds, confirmed by Hi-C data, are named according to size ( Figure 5; Table 3).

**Table 3.** Chromosomal pseudomolecules in the genome assembly of *Agelastica alni*, icAgeAlni6.

INSDC accession	Name	Length (Mb)	GC%
OX467709.1	2	86.83	34.5
OX467710.1	3	83.77	34
OX467711.1	4	67.1	34.5
OX467712.1	5	66.31	34
OX467713.1	6	54.11	34.5
OX467714.1	7	43.28	34.5
OX467715.1	8	40.27	35
OX467716.1	9	38.8	35
OX467717.1	10	35.67	35
OX467718.1	11	34.79	34
OX467719.1	12	32.21	35
OX467708.1	X	100.21	33.5
OX467720.1	MT	0.02	20.5

The mitochondrial genome was also assembled. This sequence is included as a contig in the multifasta file of the genome submission and as a standalone record.

### Assembly quality metrics

The estimated Quality Value (QV) and *k*-mer completeness metrics, along with BUSCO completeness scores, were calculated for each haplotype and the combined assembly. The QV reflects the base-level accuracy of the assembly, while *k*-mer completeness indicates the proportion of expected *k*-mers identified in the assembly. BUSCO scores provide a measure of completeness based on benchmarking universal single-copy orthologues.

The combined primary and alternate assemblies achieve an estimated QV of 56.1. The *k*-mer recovery for the primary haplotype is 96.00%, and for the alternate haplotype 81.40%; the combined primary and alternate assemblies have a *k*-mer recovery of 98.11%. BUSCO v.5.5.0 analysis using the endopterygota_odb10 reference set ( *n* = 2,124) identified 99.2% of the expected gene set (single = 97.7%, duplicated = 1.6%).

Table 2 provides assembly metric benchmarks adapted from Rhie *et al.* (2021) and the Earth BioGenome Project Report on Assembly Standards September 2024. The assembly achieves the EBP reference standard of **6.C.Q55**.

## Genome annotation report

The *Agelastica alni* genome assembly (GCA_950111635.1) was annotated by Ensembl at the European Bioinformatics Institute (EBI). This annotation includes 25,580 transcribed mRNAs from 13,498 protein-coding and 3,465 non-coding genes. The average transcript length is 16,617.90 bp. There are 1.51 coding transcripts per gene and 5.60 exons per transcript. For further information about the annotation, please refer to https://beta.ensembl.org/species/bad2c691-ca6d-47af-ace0-2a7e6df61c9f.

## Methods

### Sample acquisition and DNA barcoding

The specimen used for genome sequencing was an adult *Agelastica alni* (specimen ID NHMUK014439776, ToLID icAgeAlni6), collected from Bookham Common, Surrey, England, UK (latitude 51.29, longitude –0.39) on 2021-09-19. The specimen was collected by Danaë Vassiliades, Maxwell Barclay, Michael Geiser, Keita Matsumoto, Joana Cristóvão and Will Bayfield Farrell (Natural History Museum), identified by Michael Geiser and preserved by dry freezing (–80 °C).

Further specimens were used for Hi-C sequencing (specimen ID Ox001951, ToLID icAgeAlni4) and for RNA sequencing (specimen ID Ox001952, ToLID icAgeAlni5). These specimens were collected from Wallingford, Oxfordshire, United Kingdom (latitude 51.63, longitude –1.15) on 2021-09-16 by potting. The specimens were collected and identified by Liam Crowley (University of Oxford) and preserved on dry ice.

The initial identification was verified by an additional DNA barcoding process according to the framework developed by Twyford *et al.* (2024). A small sample was dissected from each specimen and stored in ethanol, while the remaining parts were shipped on dry ice to the Wellcome Sanger Institute (WSI) ( Pereira *et al.*, 2022). The tissue was lysed, the COI marker region was amplified by PCR, and amplicons were sequenced and compared to the BOLD database, confirming the species identification ( Crowley *et al.*, 2023). Following whole genome sequence generation, the relevant DNA barcode region was also used alongside the initial barcoding data for sample tracking at the WSI ( Twyford *et al.*, 2024). The standard operating procedures for Darwin Tree of Life barcoding have been deposited on protocols.io ( Beasley *et al.*, 2023).

Metadata collection for samples adhered to the Darwin Tree of Life project standards described by Lawniczak *et al*. (2022).

### Nucleic acid extraction

The workflow for high molecular weight (HMW) DNA extraction at the Wellcome Sanger Institute (WSI) Tree of Life Core Laboratory includes a sequence of procedures: sample preparation and homogenisation, DNA extraction, fragmentation and purification. Detailed protocols are available on protocols.io ( Denton *et al.*, 2023b). The icAgeAlni6 sample was prepared for DNA extraction by weighing and dissecting it on dry ice ( Jay *et al.*, 2023). Tissue from the thorax was homogenised using a PowerMasher II tissue disruptor ( Denton *et al.*, 2023a).

HMW DNA was extracted using the Automated MagAttract v1 protocol ( Sheerin *et al.*, 2023). DNA was sheared into an average fragment size of 12–20 kb in a Megaruptor 3 system ( Todorovic *et al.*, 2023). Sheared DNA was purified by solid-phase reversible immobilisation, using AMPure PB beads to eliminate shorter fragments and concentrate the DNA ( Strickland *et al.*, 2023). The concentration of the sheared and purified DNA was assessed using a Nanodrop spectrophotometer and a Qubit Fluorometer using the Qubit dsDNA High Sensitivity Assay kit. The fragment size distribution was evaluated by running the sample on the FemtoPulse system.

RNA was extracted from whole organism tissue of icAgeAlni5 in the Tree of Life Laboratory at the WSI using the RNA Extraction: Automated MagMax™ *mir*Vana protocol ( do Amaral *et al.*, 2023). The RNA concentration was assessed using a Nanodrop spectrophotometer and a Qubit Fluorometer using the Qubit RNA Broad-Range Assay kit. Analysis of the integrity of the RNA was done using the Agilent RNA 6000 Pico Kit and Eukaryotic Total RNA assay.

### PacBio HiFi library preparation and sequencing

Library preparation and sequencing were performed at the WSI Scientific Operations core. Samples were required to have an average fragment size exceeding 8 kb and a total mass over 400 ng to proceed to the low-input SMRTbell Prep Kit 3.0 protocol (Pacific Biosciences), depending on genome size and sequencing depth required. Libraries were prepared using the SMRTbell Prep Kit 3.0 as per the manufacturer’s instructions. The kit includes the reagents required for end repair/A-tailing, adapter ligation, post-ligation SMRTbell bead cleanup, and nuclease treatment. Size-selection and clean-up were carried out using diluted AMPure PB beads (Pacific Biosciences). DNA concentration was quantified using the Qubit Fluorometer v4.0 (ThermoFisher Scientific) with Qubit 1X dsDNA HS assay kit and the final library fragment size analysis was carried out using the Agilent Femto Pulse Automated Pulsed Field CE Instrument (Agilent Technologies) and the gDNA 55kb BAC analysis kit.

Samples were sequenced using the Sequel IIe system (Pacific Biosciences, California, USA). The concentration of the library loaded onto the Sequel IIe was in the range 40–135 pM. The SMRT link software, a PacBio web-based end-to-end workflow manager, was used to set-up and monitor the run, as well as perform primary and secondary analysis of the data upon completion.

### Hi-C data

***Sample preparation and crosslinking***

Hi-C data were generated from 20–50 mg of frozen tissue of the icAgeAlni4 sample using the Arima-HiC v2 kit (Arima Genomics). As per manufacturer’s instructions, tissue was fixed, and the DNA crosslinked using a TC buffer with a final formaldehyde concentration of 2%. The tissue was then homogenised using the Diagnocine Power Masher-II. The crosslinked DNA was digested using a restriction enzyme master mix, then biotinylated and ligated. A clean up was performed with SPRIselect beads prior to library preparation. DNA concentration was quantified using the Qubit Fluorometer v4.0 (Thermo Fisher Scientific) and Qubit HS Assay Kit, and sample biotinylation percentage was estimated using the Arima-HiC v2 QC beads.

***Hi-C library preparation and sequencing***

For Hi-C library preparation, the biotinylated DNA constructs were fragmented using a Covaris E220 sonicator and size-selected to 400–600 bp using SPRISelect beads. DNA was then enriched using Arima-HiC v2 Enrichment beads. The NEBNext Ultra II DNA Library Prep Kit (New England Biolabs) was used for end repair, A-tailing, and adapter ligation, following a modified protocol in which library preparation is carried out while the DNA remains bound to the enrichment beads. PCR amplification was performed using KAPA HiFi HotStart mix and custom dual-indexed adapters (Integrated DNA Technologies) in a 96-well plate format. Depending on sample concentration and biotinylation percentage determined at the crosslinking stage, samples were amplified for 10–16 PCR cycles. Post-PCR clean-up was carried out using SPRISelect beads. The libraries were quantified using the Accuclear Ultra High Sensitivity dsDNA Standards Assay kit (Biotium) and normalised to 10 ng/μL before sequencing. Hi-C sequencing was performed on the Illumina NovaSeq 6000.

***RNA library preparation and sequencing***

Poly(A) RNA-Seq libraries were prepared using the NEBNext ^®^ Ultra™ II Directional RNA Library Prep Kit for Illumina (New England Biolabs), following the manufacturer’s instructions. Poly(A) mRNA in the total RNA solution was isolated using oligo(dT) beads, converted to cDNA, and uniquely indexed; 14 PCR cycles were performed. Libraries were size-selected to produce fragments between 100–300 bp. Libraries were quantified, normalised, pooled to a final concentration of 2.8 nM, and diluted to 150 pM for loading. Sequencing was carried out on the Illumina NovaSeq 6000.

### Genome assembly, curation and evaluation

***Assembly***

Prior to assembly of the PacBio HiFi reads, a database of *k*-mer counts ( *k* = 31) was generated from the filtered reads using FastK. GenomeScope2 ( Ranallo-Benavidez *et al.*, 2020) was used to analyse the *k*-mer frequency distributions, providing estimates of genome size, heterozygosity, and repeat content.

The HiFi reads were first assembled using Hifiasm ( Cheng *et al.*, 2021) with the --primary option. Haplotypic duplications were identified and removed using purge_dups ( Guan *et al.*, 2020). The Hi-C reads ( Rao *et al.*, 2014) were mapped to the primary contigs using bwa-mem2 ( Vasimuddin *et al.*, 2019), and the contigs were scaffolded using YaHS ( Zhou *et al.*, 2023) using the --break option to handle potential misassemblies. The scaffolded assemblies were evaluated using Gfastats ( Formenti *et al.*, 2022), BUSCO ( Manni *et al.*, 2021) and MERQURY.FK ( Rhie *et al.*, 2020).

The mitochondrial genome was assembled using MitoHiFi ( Uliano-Silva *et al.*, 2023), which runs MitoFinder ( Allio *et al.*, 2020) and uses these annotations to select the final mitochondrial contig and to ensure the general quality of the sequence.

***Assembly curation***

The assembly was decontaminated using the Assembly Screen for Cobionts and Contaminants (ASCC) pipeline. Flat files and maps used in curation were generated via the TreeVal pipeline ( Pointon *et al.*, 2023). Manual curation was conducted primarily in PretextView ( Harry, 2022) and HiGlass ( Kerpedjiev *et al.*, 2018), with additional insights provided by JBrowse2 ( Diesh *et al.*, 2023). Scaffolds were visually inspected and corrected as described by Howe *et al*. (2021). Any identified contamination, missed joins, and mis-joins were amended, and duplicate sequences were tagged and removed. The curation process is documented at https://gitlab.com/wtsi-grit/rapid-curation.

***Assembly quality assessment***

The Merqury.FK tool ( Rhie *et al.*, 2020), run in a Singularity container ( Kurtzer *et al.*, 2017), was used to evaluate *k*-mer completeness and assembly quality for the primary and alternate haplotypes using the *k*-mer databases ( *k* = 31) computed prior to genome assembly. The analysis outputs included assembly QV scores and completeness statistics.

The genome was analysed using the BlobToolKit pipeline, a Nextflow ( Di Tommaso *et al.*, 2017) implementation of the earlier Snakemake BlobToolKit pipeline ( Challis *et al.*, 2020). The pipeline aligns PacBio reads using minimap2 ( Li, 2018) and SAMtools ( Danecek *et al.*, 2021) to generate coverage tracks. Simultaneously, it queries the GoaT database ( Challis *et al.*, 2023) to identify relevant BUSCO lineages and runs BUSCO ( Manni *et al.*, 2021). For the three domain-level BUSCO lineages, BUSCO genes are aligned to the UniProt Reference Proteomes database ( Bateman *et al.*, 2023) using DIAMOND blastp ( Buchfink *et al.*, 2021). The genome is divided into chunks based on the density of BUSCO genes from the closest taxonomic lineage, and each chunk is aligned to the UniProt Reference Proteomes database with DIAMOND blastx. Sequences without hits are chunked using seqtk and aligned to the NT database with blastn ( Altschul *et al.*, 1990). The BlobToolKit suite consolidates all outputs into a blobdir for visualisation.

The BlobToolKit pipeline was developed using nf-core tooling ( Ewels *et al.*, 2020) and MultiQC ( Ewels *et al.*, 2016), with package management via Conda and Bioconda ( Grüning *et al.*, 2018), and containerisation through Docker ( Merkel, 2014) and Singularity ( Kurtzer *et al.*, 2017).

Table 4 contains a list of relevant software tool versions and sources.

**Table 4.** Software tools: versions and sources.

Software tool	Version	Source
BEDTools	2.30.0	https://github.com/arq5x/bedtools2
BLAST	2.14.0	ftp://ftp.ncbi.nlm.nih.gov/blast/executables/blast+/
BlobToolKit	4.3.9	https://github.com/blobtoolkit/blobtoolkit
BUSCO	5.5.0	https://gitlab.com/ezlab/busco
bwa-mem2	2.2.1	https://github.com/bwa-mem2/bwa-mem2
Cooler	0.8.11	https://github.com/open2c/cooler
DIAMOND	2.1.8	https://github.com/bbuchfink/diamond
fasta_windows	0.2.4	https://github.com/tolkit/fasta_windows
FastK	666652151335353eef2fcd58880bcef5bc2928e1	https://github.com/thegenemyers/FASTK
GenomeScope2.0	2.0.1	https://github.com/tbenavi1/genomescope2.0
Gfastats	1.3.6	https://github.com/vgl-hub/gfastats
GoaT CLI	0.2.5	https://github.com/genomehubs/goat-cli
Hifiasm	0.16.1-r375	https://github.com/chhylp123/hifiasm
HiGlass	44086069ee7d4d3f6f3f0012569789ec138f42b84aa4435 7826c0b6753eb28de	https://github.com/higlass/higlass
MerquryFK	d00d98157618f4e8d1a9190026b19b471055b22e	https://github.com/thegenemyers/MERQURY.FK
Minimap2	2.24-r1122	https://github.com/lh3/minimap2
MitoHiFi	2	https://github.com/marcelauliano/MitoHiFi
MultiQC	1.14, 1.17, and 1.18	https://github.com/MultiQC/MultiQC
Nextflow	23.10.0	https://github.com/nextflow-io/nextflow
PretextView	0.2.5	https://github.com/sanger-tol/PretextView
purge_dups	1.2.3	https://github.com/dfguan/purge_dups
samtools	1.19.2	https://github.com/samtools/samtools
sanger-tol/ascc	0.1.0	https://github.com/sanger-tol/ascc
sanger-tol/ blobtoolkit	0.5.0	https://github.com/sanger-tol/blobtoolkit
Seqtk	1.3	https://github.com/lh3/seqtk
Singularity	3.9.0	https://github.com/sylabs/singularity
TreeVal	1.2.0	https://github.com/sanger-tol/treeval
YaHS	1.2a	https://github.com/c-zhou/yahs

### Wellcome Sanger Institute – Legal and Governance

The materials that have contributed to this genome note have been supplied by a Darwin Tree of Life Partner. The submission of materials by a Darwin Tree of Life Partner is subject to the **‘Darwin Tree of Life Project Sampling Code of Practice’**, which can be found in full on the Darwin Tree of Life website here. By agreeing with and signing up to the Sampling Code of Practice, the Darwin Tree of Life Partner agrees they will meet the legal and ethical requirements and standards set out within this document in respect of all samples acquired for, and supplied to, the Darwin Tree of Life Project. 

Further, the Wellcome Sanger Institute employs a process whereby due diligence is carried out proportionate to the nature of the materials themselves, and the circumstances under which they have been/are to be collected and provided for use. The purpose of this is to address and mitigate any potential legal and/or ethical implications of receipt and use of the materials as part of the research project, and to ensure that in doing so we align with best practice wherever possible. The overarching areas of consideration are:

•   Ethical review of provenance and sourcing of the material

•   Legality of collection, transfer and use (national and international) 

Each transfer of samples is further undertaken according to a Research Collaboration Agreement or Material Transfer Agreement entered into by the Darwin Tree of Life Partner, Genome Research Limited (operating as the Wellcome Sanger Institute), and in some circumstances other Darwin Tree of Life collaborators.

## Data Availability

European Nucleotide Archive: Agelastica alni (alder leaf beetle). Accession number PRJEB60008; https://identifiers.org/ena.embl/PRJEB60008. The genome sequence is released openly for reuse. The *Agelastica alni* genome sequencing initiative is part of the Darwin Tree of Life Project (PRJEB40665) and Sanger Institute Tree of Life Programme (PRJEB43745). All raw sequence data and the assembly have been deposited in INSDC databases. Raw data and assembly accession identifiers are reported in Table 1 and Table 2.

## Author information

Members of the University of Oxford and Wytham Woods Genome Acquisition Lab are listed here: https://doi.org/10.5281/zenodo.12157525.

Members of the Natural History Museum Genome Acquisition Lab are listed here: https://doi.org/10.5281/zenodo.12159242.

Members of the Darwin Tree of Life Barcoding collective are listed here: https://doi.org/10.5281/zenodo.12158331.

Members of the Wellcome Sanger Institute Tree of Life Management, Samples and Laboratory team are listed here: https://doi.org/10.5281/zenodo.12162482.

Members of Wellcome Sanger Institute Scientific Operations: Sequencing Operations are listed here: https://doi.org/10.5281/zenodo.12165051.

Members of the Wellcome Sanger Institute Tree of Life Core Informatics team are listed here: https://doi.org/10.5281/zenodo.12160324.

Members of the Tree of Life Core Informatics collective are listed here: https://doi.org/10.5281/zenodo.12205391.

Members of the Darwin Tree of Life Consortium are listed here: https://doi.org/10.5281/zenodo.4783558.
