# Supplementary material for: The genome sequence of the Alder leaf beetle, Agelastica alni (Linnaeus, 1758)
Source: Wellcome Open Res. 2025 May 20;10:263. [Version 1] doi: 10.12688/wellcomeopenres.24286.1 (PMC12586983; doi:10.12688/wellcomeopenres.24286.1)
Supplement: Peer review history

# Reviewer report: Reviewer response for version 1

**DOI:** 10.21956/wellcomeopenres.26784.r125542

**Published:** 2025-11-04

**Authors:** Arun Arumugaperumal

**Affiliations:** 1 Department of Biotechnology, Rajalakshmi Engineering College, Rajalakshmi Group of Institutions (Ringgold ID: 231560), Thandalam, Chennai 602105, Tamil Nadu, India

This data note describes the genome sequence of *Agelastica alni* also known as the Alder leaf beetle. The photograph in figure 1 helps to clearly recognize the beetle. The assembly reported here is of size 692.27 Mb size spread among 12 chromosomes. The mitogenome of size 17.39 kb was also sequenced. The taxonomy was clearly mentioned. It would be appropriate to mention the X0 chromosome system otherwise readers will be confused whether this a male or female specimen. A BUSCO score of 99% indicates the completeness of the genome as per database mandates.

Are sufficient details of methods and materials provided to allow replication by others?

Yes

Is the rationale for creating the dataset(s) clearly described?

Yes

Are the datasets clearly presented in a useable and accessible format?

Yes

Are the protocols appropriate and is the work technically sound?

Yes

Reviewer Expertise:

Bioinformatics; Genomics

I confirm that I have read this submission and believe that I have an appropriate level of expertise to confirm that it is of an acceptable scientific standard.

# Reviewer report: Reviewer response for version 1

**DOI:** 10.21956/wellcomeopenres.26784.r124799

**Published:** 2025-06-17

**Authors:** Henrique Antoniolli

**Affiliations:** 1 Universidade Federal do Rio Grande do Sul (UFRGS), Porto Alegre, Brazil

The present study reports a chromosome-level assembly and the mitochondrial genome of the alder leaf beetle *Agelastica alni*. Overall, the report is well written, providing information about the beetle’s general biology and the motivation for sequencing its genome. Accession numbers for each generated dataset and the versions of the software used are properly documented. The assembly achieved satisfactory levels of completeness.

 Minor issue:

- Why was the mitochondrial genome not gene-predicted and annotated?

Are sufficient details of methods and materials provided to allow replication by others?

Yes

Is the rationale for creating the dataset(s) clearly described?

Yes

Are the datasets clearly presented in a useable and accessible format?

Yes

Are the protocols appropriate and is the work technically sound?

Yes

Reviewer Expertise:

Genomics, evolution

I confirm that I have read this submission and believe that I have an appropriate level of expertise to confirm that it is of an acceptable scientific standard.
